# The Aim Justifies the Means—Differences Among Musical and Nonmusical Means of Relaxation or Activation Induction in Daily Life

**DOI:** 10.3389/fnhum.2019.00036

**Published:** 2019-02-22

**Authors:** Mattes B. Kappert, Alexandra Wuttke-Linnemann, Wolff Schlotz, Urs M. Nater

**Affiliations:** ^1^Clinical Biopsychology, Department of Psychology, University of Marburg, Marburg, Germany; ^2^Center for Mental Health in Old Age, Landeskrankenhaus (AöR), University Medical Centre Mainz, Department of Psychiatry and Psychotherapy, Mainz, Germany; ^3^Max Planck Institute for Empirical Aesthetics, Frankfurt am Main, Germany; ^4^Division of Clinical Psychology, Department of Psychology, University of Vienna, Vienna, Austria

**Keywords:** ambulatory assessment, autonomic nervous system, ecological momentary intervention, music, relaxation, stress, stress management, stress reduction

## Abstract

Music is an effective means of stress-reduction. However, to date there has been no systematic comparison between musical and language-based means of stress reduction in an ambulatory setting. Furthermore, although the aim for listening to music appears to play a role in its effect, this has not yet been investigated thoroughly. We compared musical means, language-based means like guided relaxation or self-enhancement exercises, and a combination of both with respect to their potential to reduce perceived stress. Furthermore, we investigated whether the aim one wants to achieve by listening to these means had an impact on their effect. We tested 64 participants (age: *M* = 40.09 years; 18 female) for 3–10 days during their everyday life using an app containing three means: musical means, language-based means, and a combination of both. For the music and the combination conditions participants were asked to select an aim: relaxation or activation. We measured perceived stress, relaxation, activation, and electrical skin resistance (ESR) as a marker of sympathetic nervous system (SNS) activity before and after using the app. Participants were instructed to use the app as often as desired. Overall, perceived stress was reduced after using the app, while perceived relaxation and activation were increased. There were no differences between the three means regarding their effect on perceived stress and relaxation, but music led to a greater increase in ESR and perceived activation compared to the other means. There was a decrease in ESR only for music. Moreover, perceived stress was reduced and perceived relaxation was increased to greater extent if the aim “relaxation” had been selected. Perceived activation, however, showed a larger increase if the aim had been “activation,” which was even more marked in the case of music listening. Our results indicate that all three means reduced perceived stress and promoted feelings of relaxation and activation. For enhancing feelings of activation music seems to be more effective than the other means, which was reflected in increased SNS activity as well. Furthermore, the choice of an aim plays an important role for the reduction of stress, and promotion of relaxation and activation.

## Introduction

Stress is a potential health threat (McEwen, 1998). While short-term stress is harmless and adaptive, chronic stress is assumed to be detrimental and to be involved in the genesis and exacerbation of conditions such as myocardial infarction, diabetes, asthma or gastrointestinal diseases (McEwen and Stellar, 1993). Stress is not a mere subjective phenomenon, but affects several physiological systems such as the autonomic nervous system (ANS), which is involved in the body’s response to a stressor. The ANS consists of two subsystems: the parasympathetic nervous system (PNS), which stimulates rest and restoration of the body’s energy resources, and the sympathetic nervous system (SNS), which sets energy free and stimulates the body’s fight-or-flight response. Even though an increase of SNS activity occurs as a normal correlate of activation of the organism, high SNS activity may also indicate the experience of stress.

A major source of stress in modern-day life is work and the growing demands it poses. A survey by the The American Institute of Stress (2014) found that job pressure was the most important cause of stress in the United States. Similar findings have been reported in numerous other countries, such as Great Britain (Health and Safety Executive, 2017) or Germany (Techniker Krankenkasse, 2016). In view of these high stress burdens and the aforementioned hazards of long-term stress, there is a great demand for effective and feasible means of stress reduction.

Various methods have been shown to reduce stress, including guided relaxation, or meditation techniques (Goyal et al., 2014) or yoga (Sharma, 2014). Moreover, methods of activation such as exercise are also used to cope with stress. Interestingly, exercise seems to reduce feelings of tension and perceived stress even though acute bouts of exercise may induce a physiological stress response (Strahler et al., 2017). Unfortunately, some of these techniques are time-consuming (like yoga and exercise) while others demand a certain level of training in order to be effective (like relaxation techniques and meditation). This might limit their applicability in certain domains of everyday life, particularly during work.

Another means of stress reduction is listening to music (Pelletier, 2004; Thoma and Nater, 2011). Listening to music is fairly common in everyday life (North et al., 2004). Since music costs little, has hardly any adverse side effects, and is easily accessible, for instance *via* MP3 players or smartphones (Skånland, 2012), it is a valuable tool for reducing stress in everyday life and might therefore be particularly suitable for use in work settings. Over the last years, our group has conducted several ecological momentary assessment (EMA) studies to investigate the beneficial effects of listening to music (Linnemann et al., 2015a,b, 2016). These studies showed that music has an impact on reports of subjective stress as well as on markers of ANS activity. Which music is being selected by an individual in a specific situation has been shown to be associated with the function that music serves for an individual in that specific situation (Greb et al., 2018a,b). Extending this notion to effects of music listening, we found that the reason for listening to music determined its stress reducing effect: listening to music for “relaxation” was associated with decreases in perceived stress levels (Linnemann et al., 2015a, 2016) as well as lower salivary cortisol levels (Linnemann et al., 2015a) whereas other reasons like “reducing boredom” or “distraction” were not (Linnemann et al., 2015a,b, 2016). In one of the studies (Linnemann et al., 2015b), listening to music for the reason of activation was associated with a reduction in perceived stress, corroborating the notion that activating and arousing activities may also be useful in order to reduce stress (as is the case with exercising), even though this might seem counterintuitive at first glance. However, the aforementioned studies investigated the association between music listening and measures of stress at least partially in a retrospective manner. In other words, at given time points, participants were asked to indicate their music listening behavior within the last hours and their perceived stress at the time of measurement, leaving a time lag between the event and its measurement. Although the retrospection period was short, it might induce bias into measurements. Therefore, it would be valuable to conduct a prospective intervention-like study, in which participants are asked to listen to music and state their reasons for listening or the aims they wish to achieve by it with measurements of stress conducted directly before and after listening.

Clearly, however, music is not the only cost-efficient and easily accessible means to achieve stress-reduction. Language-based means such as guided relaxation exercises are similarly accessible and cost-efficient, giving rise to the question whether musical and language-based means are equally effective in terms of reducing stress. Unfortunately, studies comparing these means are sparse, although there are some studies comparing musical and non-musical means with regard to their effects on ANS parameters, which can be seen as suitable markers of stress. Most of these studies did not find differences between musical and language-based means in terms of their effect on parameters such as finger temperature or heart rate (Kibler and Rider, 1983; Scheufele, 2000). Schilling and Poppen (1983) found a main effect of group when comparing several different non-musical and one musical means with regard to skin conductance level, but *post hoc* tests were not significant. In contrast, Jing et al. (2011) found that guided imagery led to a lower heart rate than did music, albeit for the rather specific situation of centrifuge training for future astronauts. However, the latter study found no differences in heart rate immediately after centrifuge training and no differences in heart rate variability parameters. Overall, the findings are somewhat inconclusive, but tend to hint at comparable effects of certain musical and language-based means. Nevertheless, some important issues have not yet been addressed.

First, the aforementioned studies only investigated relaxing music or relaxation exercises and did not take into account means that might be perceived as activating or arousing, even though both relaxation and activation seem to be common and important functions of listening to music (North and Hargreaves, 2000; Greb et al., 2018b). Second, these studies compared only one musical and a limited selection of non-musical means of relaxation (and their combination in the study by Kibler and Rider, 1983), raising questions about the generalizability of the findings with respect to other music or relaxation techniques. Thus, there is a need to compare a greater variety of musical and non-musical means, both relaxing and activating. Third, as the previous studies were conducted in laboratory settings, it is unclear whether the findings can be applied to everyday life settings as well. Using ambulatory assessment methods could provide data from a more naturalistic context leading to higher ecological validity of the findings. Finally, most of the samples consisted of students (with the exception of Schilling and Poppen, 1983). Thus, it might be useful to investigate whether the aforementioned findings can be replicated in a working population with high job demands.

In order to thoroughly test the comparability of musical and non-musical relaxation techniques and their effects on stress, taking into account the shortcomings mentioned above, the present study pursued the following aims: we compared the effects of: (1) music; (2) guided relaxation or self-enhancement exercises accompanied by music; and (3) merely language-based relaxation and self-enhancement instructions, with regard to their effects on perceived stress and on SNS activity. A variety of stimuli were used and the study was conducted in a daily life setting using ambulatory assessment methods and a sample of participants with demanding job profiles and high stress levels. Furthermore, taking into account the aforementioned findings on the role of functions of listening to music, we additionally tested the effects of aims for listening to music.

## Materials and Methods

### Participants

A total of 70 participants were recruited for the study *via* a market research institute in three major German cities (Berlin, Frankfurt am Main, Munich). All participants worked in elevated positions, such as managers, entrepreneurs or higher public officials. The eligibility criteria were as follows: age between 30 and 50 years, fluency in the German language, travel for professional reasons at least once per month, work in elevated positions, and ability to use the app (see below) at least once per day. Two participants were excluded from the data analyses because they did not provide demographic data and four were excluded because the app data were missing due to technical defects. The final sample thus consisted of 64 participants (18 female; mean age: 40.09 ± 6.36 years).

### Procedure

Our investigation was embedded in an industry-funded study whose aim was to test an app designed to reduce stress and promote relaxation and activation *via* musical and non-musical means. Upon meeting eligibility criteria participants were invited to a branch of a market research institute in one of the aforementioned cities (based on their home address), where they were provided with “Momentum 2” over-ear headphones (Sennheiser, Wedemark, Germany) and an iPod 5^®^ (Apple, Cupertino, USA). The iPod 5^®^ was installed with an app containing a purely musical program, a combined musical and language-based program, and a purely language-based program (see section “Momentary Assessment”). Participants also received a device for measuring their electrical skin resistance (ESR), which was connected to the iPod *via* Bluetooth. Instructions on how to use the app and the device for measuring ESR were provided by video tutorial, and participants also received a manual for handling the iPod, the app and the device for measuring ESR. Participants were instructed to use the app as often as desired, but to use it at least once per day, if possible. The mean frequency of uses per day was 1.38 times (SD = 0.66). Since participants worked in elevated positions with high work demands, they were given the opportunity to choose the duration of their participation from 3 to 10 days (mean 7.31 days, SD = 1.36) in order to keep the burden due to study participation at a tolerable level. Upon completing the measurement period, they returned to the respective branch of the market research institute, handed back the devices, and were asked to complete an online survey containing psychological questionnaires as well as questions about demographic data. This study was carried out in accordance with the recommendations of the German Psychological Society. All participants gave written informed consent in accordance with the Declaration of Helsinki. As a comparable protocol (Linnemann et al., 2018) had already been approved by the Local Ethics Committee of the University of Marburg, no specific ethical approval was sought for the current protocol. Participants received a compensation of 240 € for their participation.

### Momentary Assessment

Our methodological approach was similar to EMA (Stone and Shiffman, 1994). According to Shiffman et al. (2008), EMA subsumes approaches in which data are collected repeatedly over time, in real-world settings, and with a focus on participants’ current state rather than longer retrospective recall. EMA can include various designs with respect to the assessment times: assessment can take place when a particular behavior of interest occurs or at random time points. Our approach comprises assessment before and after using one of three means of relaxation or activation, as was done similarly by e.g., Randall and Rickard (2017). However, in our study participants were not merely asked to provide data whenever they used the abovementioned means, but they were rather asked to actively use these means. This means our approach can be regarded as an intervention to a certain extent. Therefore, our method can best be described as a combination of EMA and ecological momentary intervention (EMI; Heron and Smyth, 2010). Yet, a noteworthy difference to many EMIs is that participants in our study could choose time, duration and nature (musical, language-based or combined means) of the intervention themselves.

We used a non-commercially available app (Nerdindustries GmbH, Hamburg, Germany) to compare musical and non-musical methods of relaxation and activation. The app was designed to promote relaxation or activation or to prepare individuals for certain professional situations using musical or language-based means, or a combination of both. It could be used in three different modes: (1) listening to music only (“music”); (2) listening to guided relaxation or self-enhancement exercises accompanied by music (“music and language”); or (3) listening to relaxation and self-enhancement exercises without any musical elements (“language”). Participants had different options to customize their sessions, depending on the mode they used: in modes 1 and 2 participants could select one of two aims for listening: relaxation or activation. Furthermore, participants could select a duration of either 5, 10, 15 or 20 min, and could choose a musical genre: Rock, Pop, and Classical in mode 1, and Ambient, Lounge or Acoustic in mode 2. Participants were free to choose when and how to use the app. Every time they started a session, they could select a mode, an aim, a duration or a genre as desired. They could also decide at will at what time of the day to use the app. Moreover, it was possible to end a session at any time. Mode 1 contained a variety of popular pieces of music in the English or German language as well as instrumental pieces. The app provided a specific set of songs depending on participants’ choice of aim and genre. Songs could be skipped if desired, which resulted in the next song in the set list being played. The session ended once the previously selected time had expired. Mode 2 contained a variety of different guided relaxation or activation tracks, accompanied by instrumental music. The provided tracks and background music depended on the choice of aim. Tracks could be skipped if desired, which resulted in another track being played. The session ended once the previously selected time had expired. In Mode 3 participants could choose a specific track, suited for preparation for a certain situation (e.g., before an important presentation or other challenging situations, or relaxation instructions). The session ended when the entire track had been listened to. The duration of the tracks in this mode ranged from 249 to 629 s. Before and after every session, participants were asked to rate their perceived stress, relaxation and activation. Additionally, they measured their ESR before and after each session. For each session, it was controlled whether the participant had listened to the selected track for at least 3 min. Otherwise, we assumed that the session had been too short to have an effect and excluded it from the analyses.

### Measures

#### Measures of Stress, Relaxation and Activation

In order to keep the burden for participants as low as possible, we used a one-item approach for all measures within the app in line with Elo et al. (2003). Perceived stress was measured using the statement “At the moment I feel stressed,” which was rated on a 5-point Likert scale ranging from “not at all” (0) to “very much” (4). To capture other facets of stress, we also asked participants to rate their perceived relaxation. Moreover, we asked participants to rate their perceived activation. To investigate the extent to which participants were able to replenish their energy and feel refreshed (“activated”) by using the app, we used appropriate items from a short scale based on the Multidimensional Mood Questionnaire by Wilhelm and Schoebi (2007), but altered the response scale from a 7-point to a 5-point Likert scale. To measure perceived relaxation, participants were shown the statement “At the moment I feel…,” and were asked to rate how they felt on a 5-point Likert scale from “relaxed” (0) to “tense” (4). For easier interpretation we recoded the values so that high values indicated a high degree of relaxation. To assess perceived activation, participants were likewise shown the sentence “At the moment I feel…,” and rated how they felt on a 5-point Likert scale from “without energy” (0) to “energized” (4). Chronic stress was measured using the 12-item Chronic Stress Screening Scale (CSSS; Schulz et al., 2004). On this scale, respondents indicate the frequency of stressful experiences over the last 3 months on a 5-point Likert scale ranging from “never” (1) to “very often” (5). The psychometric properties of the CSSS are good, with an internal consistency of *α* = 0.87 (Schulz et al., 2004).

#### Measure of Sympathetic Nervous System Activity

ESR was measured as a marker of SNS activity. We used a Seeedstudios Grove GSR-Sensor (Seeed Development Limited, Shenzhen, People’s Republic of China), which had been built into a plastic case with nickel electrodes placed on the outside. The sensor had an input voltage of 5V/3.3V and recorded with a sampling rate of 15 Hz. It was connected to the app *via* Bluetooth. Participants put the tips of their index and middle fingers of their preferred hand onto the electrodes. When the app registered a change in electrical resistance of at least 10%, it was assumed that the fingers had been placed on the electrodes and recording was started. Then, ESR was recorded for 10 s. If participants removed their fingers before completion of the measurement, a notification appeared in the app, asking them to place their fingers onto the electrodes again until a total of 10 s of ESR measurement had been recorded. Thereafter, the median of the measured values was translated into values ranging from 0 (low resistance) to 1,024 (high resistance). We chose to use the median rather than the mean because the latter is more susceptible to distortion due to outliers.

### Data Analysis

All analyses were carried out using SPSS^®^ Statistics 24 (IBM). Differences between the assessment sites concerning age and chronic stress were tested *via* univariate analyses of covariance. When age was the dependent variable, chronic stress and sex were included as covariates, whereas when chronic stress was the dependent variable sex and age were selected as covariates. Differences between the assessment sites regarding sex were tested *via* Chi-square test of independance. We used linear mixed-effects regression models to test the influence of modes and aims on perceived stress, relaxation and activation as well as ESR. For each of the outcomes we calculated two models. Model 1 included data of all sessions but without the independent variable “aim.” Model 2 contained both “mode” and “aim” as well as their interaction term as independent variables excluding all sessions that had been conducted in mode 3 (as in mode 3 participants did not have the opportunity to choose an aim). Furthermore, a variable for measurement time (before or after using the app) was included in all models. Apart from these variables of interest, we added several control variables to the models: participants’ sex, age, and chronic stress measured *via* the CSSS, as well as the duration of sessions, the time of day in hours during which a session was initiated, and a variable indicating whether a session took place on a weekday or at the weekend. All continuous variables (age, chronic stress measured *via* the CSSS and duration of the session) were grand-mean-centered around zero, except for the variable that indicated the time of the day when the session had taken place; this variable was grand-mean-centered around 07:00 a.m. as an approximate awakening time. All variables were entered into the fixed part of the model. Random intercepts were estimated for all participants and measurement times (with regard to before or after using the app) within participants in order to account for the nested data structure. Parameters were estimated using restricted maximum likelihood. Pairwise comparisons based on estimated marginal means (EMM) were used to determine direction and extent of the differences between categorical predictors. These comparisons were adjusted for multiple comparisons using Sidak’s method. *P-values* of ≤0.05 were considered significant.

For ESR data, extreme values were identified using boxplots and subsequently excluded because they were likely to be caused by artifacts (such as movement or insufficient contact of skin with the sensors). For psychological measures, however, outliers or extreme values were not excluded because all possible values were theoretically meaningful and exclusion would have resulted in distorted data.

## Results

### Sociodemographic Data

Data of 64 participants and 642 sessions were available for analysis. The final sample consisted of 18 women and 46 men, with a mean age of 40.09 years (SD = 6.36). The mean CSSS score was 31.64 points (SD = 8.92), indicating a high stress burden compared to norm values for the respective age group. Thirty-two of the participants (50.0%) were unmarried, 28 (43.8%) were married, and four (6.3%) were divorced. Most participants had acquired a high level of education: 50 (78.1%) had a degree from a university or a university of applied sciences, seven (10.9%) had graduated from vocational/master craftsman school, six (9.4%) had completed dual vocational training and one (1.6%) had received another education, which was not further specified. Thirty-six participants were recruited from Berlin, 15 from Munich and 13 from Frankfurt. The participants from these three cities did not differ regarding age (*F*_(2,61)_ = 0.036, *p* = 0.965), sex ($\chi_{\left(2\right)}^{2}$ = 0.063, *p* = 0.969), or chronic stress (*F*_(2,61)_ = 0.122, *p* = 0.885).

The final sample used in the analyses of ESR consisted of 63 participants (17 women, 46 men; mean age = 40.13 ± 6.41). One participant had only produced extreme values in the ESR measurements and was thus eliminated completely from ESR analyses. A final sample of 566 sessions was considered for analysis.

### Use of the App

The app was used 431 times (67.2%) in mode 1 (“music”), 114 times (17.8%) in mode 2 (“music and language”) and 96 times (15.0%) in mode 3 (“language”), indicating that listening to music alone was the most popular choice. The two aims were selected similarly often, with “relaxation” being selected 286 times (52.5%) and “activation” being selected 256 times (47.5%). Within the mode “music,” “relaxation” was selected 220 times (51.0%) and “activation” 211 times (49.0%). Within “music and language” “relaxation” was selected 66 times (57.9%) and “activation” 48 times (42.1%).

### Differences in Stress, Activation, and ESR Based on Time Point (Before vs. After Use of the App) and Mode

We examined whether perceived stress, relaxation, activation and ESR changed from before to after using the app and whether the changes differed between the modes. For each of the outcomes we calculated a model as specified in section “Data Analysis.” Only the dependent variables differed between the models. Sessions from all modes were included in the analyses.

**Figure 1:**
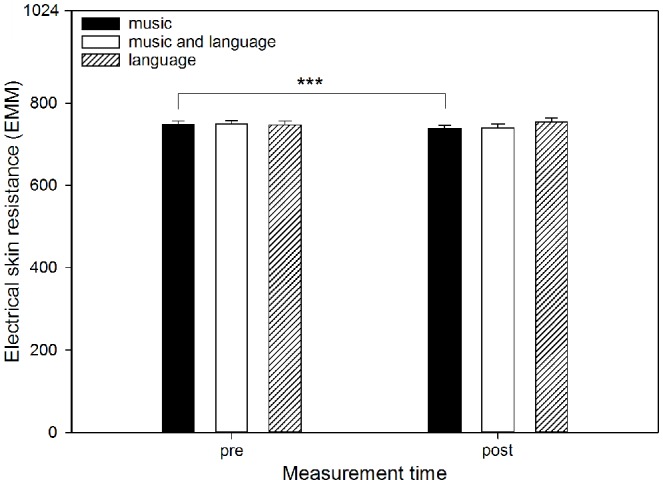
Electrical skin resistance (ESR) by listening mode before and after using the app. The graph shows the estimated marginal means (EMM) of ESR before (pre) and after (post) using the app, separated by the three modes (“music,” “music and language” and “language”). Error bars represent standard errors of EMM. ***p < 0.001.

**Figure 2:**
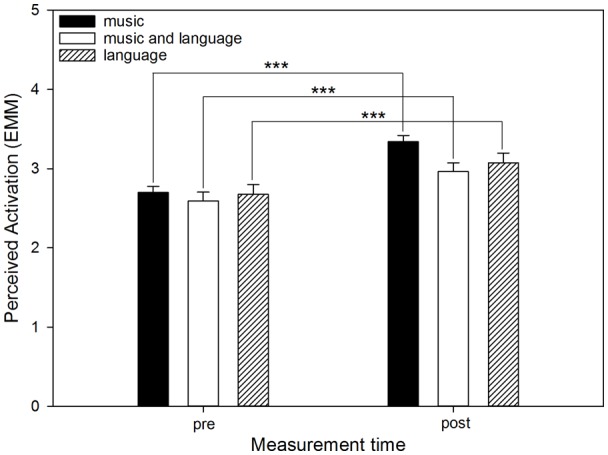
Perceived activation by listening mode before and after using the app. The graph shows the EMM of perceived activation before (pre) and after (post) using the app, separated by the three modes (“music,” “music and language” and “language”). Error bars represent standard errors of EMM. There was a significant increase from before to after using the app when all sessions independently of the mode were taken into account. However, this increase only came about due to a significant increase in the mode “music.” ***p < 0.001.

Perceived stress (*F*_(1,636)_ = 34.14, *p* < 0.001) decreased from before to after using the app, whereas perceived relaxation (*F*_(1,636)_ = 61.06, *p* < 0.001) and perceived activation (*F*_(1,636)_ = 75.19, *p* < 0.001) increased. ESR did not change significantly (*F*_(2,569.17)_ = 2.03, *p* = 0.154). Non-significant interactions of “measurement time” and “mode” revealed that the mode had no significant effect on the change in perceived stress (*F*_(2,636)_ = 1.71, *p* = 0.182) or perceived relaxation (*F*_(2,636)_ = 1.21, *p* = 0.300) from before to after using the app. However, significant interactions emerged for ESR (*F*_(2,568.97)_ = 4.16, *p* = 0.016; see Figure 1) and perceived activation (*F*_(2,636)_ = 3.87, *p* = 0.021; see Figure 2). For perceived activation, *post hoc* tests based on EMM did not reveal differences between the modes before using the app (“music” vs. “music and language”: mean difference (MD) = 0.11, *p* = 0.680; “music” vs. “language”: MD = 0.02, *p* = 0.997; “music” and language’ vs. “language”: MD = −0.09, *p* = 0.907), but after using the app, perceived activation was higher if “music” had been selected as the mode compared to “music and language” (MD = 0.38, *p* = 0.001). Perceived activation was marginally higher for “music” than for “language” (MD = 0.27, *p* = 0.066), whereas “music and language” and “language” (MD = −0.11, *p* = 0.439) did not differ significantly. Furthermore, the *post hoc* tests based on EMM revealed significant increases in perceived activation from before to after using the app within all modes (“music”: MD = −0.64, *p* < 0.001; “music and language”: MD = −0.37, *p* < 0.001; “language”: MD = −0.40, *p* < 0.001). Descriptively, this increase was somewhat more pronounced if “music” had been selected. For ESR, the *post hoc* tests based on EMM yielded no differences between the modes before using the app (“music” vs. “music and language”: MD = −0.21, *p* > 0.999; “music” vs. “language”: MD = 1.88, *p* = 0.991; “music and language” vs. “language”: MD = 2.10, *p* = 0.993), but after using the app, there was a trend towards a difference between “music” and “language” (MD = −16.10, *p* = 0.075). There were no significant differences between “music” and “music and language” (MD = 1.81, *p* = 0.990) or between “music and language” and “language”: MD = −14.30, *p* = 0.272). Furthermore, *post hoc* tests based on EMM revealed a significant decrease in ESR from before to after using the app only in the mode “music” (MD = 10.40, *p* < 0.001), whereas no such decreases emerged for “music and language” (MD = 8.81, *p* = 0.091) or “language” (MD = −7.59, *p* = 0.181).

### Differences Between Aims

We examined whether the changes in perceived stress, relaxation and activation, as well as in ESR, from before to after using the app differed between the modes “music” and “music and language” and between the aims “relaxation” and “activation,” and whether the interaction between these variables had an impact. For each of the outcomes, we calculated a model as specified in section “Data Analysis.” Only the dependent variables differed between the models. In all of the following models, sessions in the mode “language” were excluded because this mode did not provide a choice of aim.

**Figure 3:**
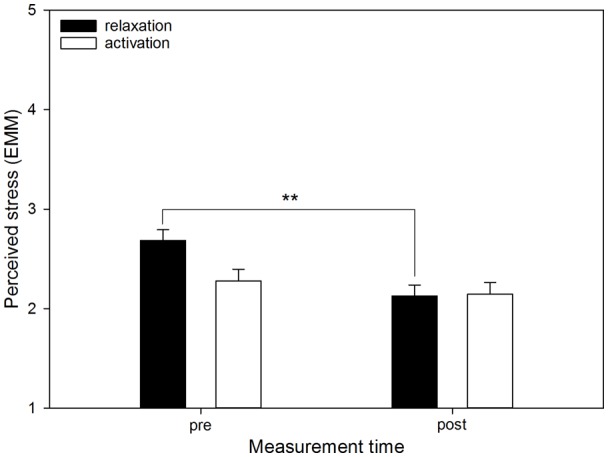
Perceived stress by listening aim before and after using the app. The graph shows the EMM of perceived stress before (pre) and after (post) using the app, separated by the two aims (“relaxation,” “activation”). Error bars represent standard errors of EMM. There was a significant increase from before to after using the app only if “relaxation” had been selected as the aim. Sessions conducted in the mode “language” were not included in the data, because in this mode there was no option of selecting an aim. **p < 0.01.

**Figure 4:**
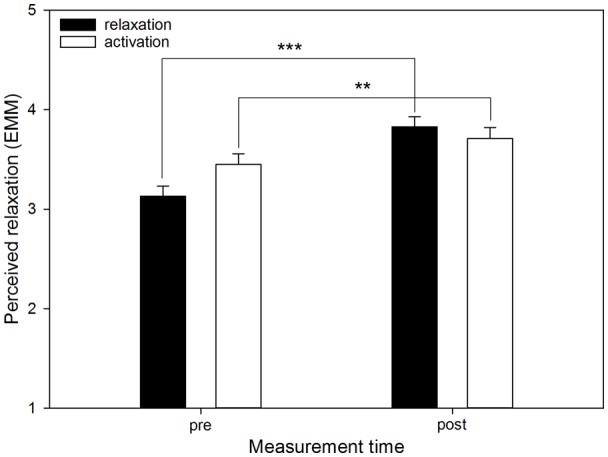
Perceived relaxation by listening aim before and after using the app. The graph shows the EMM of perceived relaxation before (pre) and after (post) using the app, separated by the two aims (“relaxation,” “activation”). Error bars represent standard errors of EMM. There were significant increases if “relaxation” and “activation” had been selected as the aim. However, the increase for the aim relaxation was clearer. Sessions conducted in the mode “language” were not included in the data, because in this mode there was no option of selecting an aim. **p < 0.01, ***p < 0.001.

**Figure 5:**
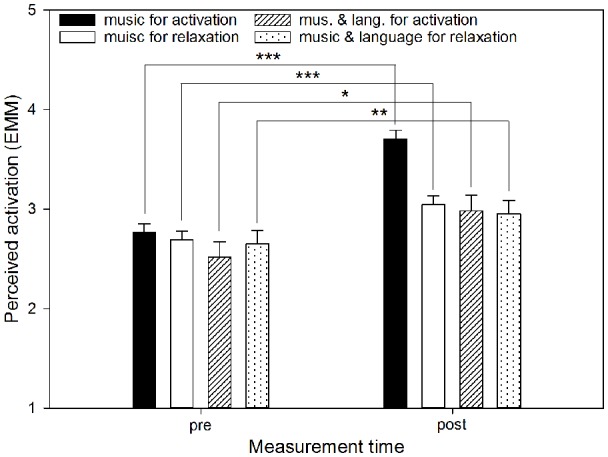
Perceived activation by listening mode and aim before and after using the app. The graph shows the EMM of perceived activation before (pre) and after (post) using the app, separated by the modes “music” and “music and language” as well as the two aims (“relaxation,” “activation”). Error bars represent standard errors of EMM. All of the combinations of aim and mode led to significant increases in perceived activation, yet the increase was most marked if music had been listened to for the aim of activation. Sessions conducted in the mode “language” were not included in the data, because in this mode there was no option of selecting an aim. *p < 0.05, **p < 0.01, ***p < 0.001.

There were significant interactions between “measurement time” and “aim” for perceived stress (*F*_(1,539)_ = 14.92, *p* < 0.001; see Figure 3), perceived relaxation (*F*_(1,539)_ = 13.14, *p* < 0.001; see Figure 4) and perceived activation (*F*_(1,539)_ = 10.37, *p* = 0.001) but not for ESR (*F*_(1,484.38)_ = 0.94, *p* = 0.332). Furthermore, for perceived activation, there was a significant interaction of “measurement time” and “mode” (*F*_(1,539)_ = 5.06, *p* = 0.025), and of “measurement time,” “mode” and “aim” (*F*_(2,531.68)_ = 4.51, *p* = 0.011; see Figure 5). For the other outcomes neither the former (perceived stress: *F*_(1,539)_ = 0.15, *p* = 0.701; perceived relaxation: *F*_(1,539)_ = 0.62, *p* = 0.431; ESR: *F*_(1,438.95)_ = 0.09, *p* = 0.768) nor the latter interaction (perceived stress: *F*_(2,526.08)_ = 1.18, *p* = 0.308; perceived relaxation: *F*_(2,528.75)_ = 0.17, *p* = 0.847; ESR: *F*_(2,474.63)_ = 0.358, *p* = 0.699) were significant.

*Post hoc* comparisons based on EMM revealed that perceived stress before using the app was higher if the aim “relaxation” had been chosen compared to the aim “activation” (MD = 0.41, *p* = 0.001). However, there was no difference in stress between the aims after using the app (MD = −0.02, *p* = 0.879). Furthermore, if “relaxation” had been selected, there was a significant decrease in perceived stress from before to after using the app (MD = 0.56, *p* < 0.001), which was not the case if “activation” had been chosen (MD = 0.13, *p* = 0.109). For perceived relaxation, *post hoc* comparisons based on EMM showed that before using the app, perceived relaxation was lower if the aim “relaxation” had been selected compared to the aim “activation” (MD = −0.32, *p* = 0.005), whereas there was no difference between the aims after using the app (MD = 0.12, *p* = 0.302). Perceived relaxation increased significantly from before to after using the app if “relaxation” (MD = −0.70, *p* < 0.001) as well as “activation” (MD = −0.26, *p* = 0.004) had been selected as the aim, but this increase was more marked if “relaxation” had been the aim. For perceived activation, *post hoc* tests based on EMM showed that before using the app, and if the aim “relaxation” had been selected, there was no difference between “music” and “music and language” (MD = 0.04, *p* = 0.773) with regard to perceived activation. There was also no difference if “activation” had been selected as the aim (MD = 0.25, *p* = 0.127). After using the app, however, and if “activation” had been selected as the aim, having selected the mode “music” resulted in greater perceived activation compared to “music and language” (MD = 0.72, *p* < 0.001), whereas the two modes did not differ if “relaxation” had been chosen as the aim (MD = 0.09, *p* = 0.510). Moreover, within the mode “music” significant increases in perceived activation emerged from before to after using the app if either “relaxation” (MD = −0.36, *p* < 0.001) or “activation” (MD = −0.94, *p* < 0.001) had been selected as the aim, but descriptively this increase was more marked if “activation” had been selected as the aim. The same was true within the mode “music and language,” where there were also significant increases from before to after using the app if “relaxation” (MD = −0.30, *p* = 0.025) or “activation” (MD = −0.47, *p* = 0.004) had been the aim, but again, selecting the aim “activation” resulted in a descriptively more pronounced increase. Finally, the tests revealed that within the mode “music,” there was no difference between the aims before using the app (MD = −0.07, *p* = 0.474), but after using the app, perceived activation was greater if the aim “activation” had been selected (MD = −0.66, *p* < 0.001). Within the mode “music and language,” however, there were no differences between the aims before (MD = −0.14, *p* = 0.480) or after using the app (MD = −0.03, *p* = 0.875).

## Discussion

### Summary of Results

Three main findings were obtained: (1) there were no differences between music, language based-means, and a combination of both with regard to reducing stress or increasing relaxation, but music was more effective than a combination of music and language-based means in promoting feelings of activation (and marginally more effective than mere language-based means). Furthermore, only music also led to a decrease in ESR, indicating a rise in SNS activity; (2) the aim of listening to music or a combined musical and language-based means played an important role: perceived stress was only reduced if participants had used the app with the aim to relax. Moreover, if the app was used in order to relax, perceived relaxation increased to a greater extent compared to the aim “activation”; and (3) perceived activation however, increased most if music had been listened to for achieving activation. This combination of mode and aim led to the most pronounced increase from before to after using the app compared to the other combinations.

In addition, we found that music was selected far more often than language-based methods or the combination of both.

### Stress Reducing Effects of Musical Compared to Language-Based or Combined Means

The current results reveal that music is equally effective in reducing perceived stress and increasing perceived relaxation as language-based techniques and a combination of both. Regarding the effect on perceived activation, however, music worked better than the combination of music and language and marginally better than mere language-based means. Thus, if one’s aim is to become energized, music alone might be a somewhat superior choice. ESR only decreased if participants had listened to music; it did not decrease if they had listened to language-based means or a combination of both. Accordingly, listening to music led to an increase in sympathetic activity. This finding is in contrast to for example Kibler and Rider (1983), who found increases in PNS activity and perceived relaxation for music and for a language-based relaxation exercise as well as the combination of both. Nevertheless, there are other studies showing an increase in SNS activity or a decrease in PNS activity when listening to music (Dillman Carpentier and Potter, 2007; Hilz et al., 2014). Whether music has a relaxing or arousing effect depends on certain musical characteristics like tempo, rhythm and emotions induced by music (Koelsch, 2014). Therefore, in future studies it might be helpful to control for these factors in order to determine how different states of psychophysiological arousal are elicited. Furthermore, it is noteworthy that an increase in SNS activity does not necessarily represent an increase in stress but can also be a result of physiological arousal as a consequence of happiness or various other emotional states elicited by music (e.g., Khalfa et al., 2002). Thus, the decrease in ESR we found might also reflect unspecific arousal, which is in line with the finding that there was a general increase of perceived activation after using the app, which was especially marked if participants had listened to music as compared to combined musical and language-based means, and marginally greater compared to merely language-based means. In order to fully cover the wide range of biological stress markers and thus to comprehensively assess stress-related activity (see Nater, 2018), future studies should employ additional biological markers such as salivary cortisol.

In addition to being more effective in elevating activation, music was the most popular choice, as it was selected far more often than language or the combination of music and language. This might hint at a somewhat higher applicability of music as a means of stress reduction in everyday life.

### The Role of the Aim

The studies by Linnemann and colleagues yielded somewhat inconsistent results with two studies finding that stress was reduced if music had been listened to for the reason of relaxation (Linnemann et al., 2015a, 2016) and one study demonstrating this effect if music had been listened to for the reason of activation (Linnemann et al., 2015b). This discrepancy in findings might be explained by the different study populations (students vs. patients with fibromyalgia syndrome, characterized by dysregulations in stress-sensitive systems), potentially meaning that, depending on the experience of stress, the effects of music listening vary. In the current study in which highly stressed individuals working in elevated positions were investigated, we found that overall, listening to music, language-based means or a combination of both was effective in reducing perceived stress and in promoting relaxation and activation. However, when taking into account the aims, it emerged that perceived stress only decreased if music had been listened to for the aim of relaxation, but not if it had been listened to in order to achieve activation. Both aims led to significant increases in perceived relaxation and activation, but the extent to which the outcomes changed from before to after using the app differed between the two aims: when the aim was to relax, perceived relaxation increased to greater extent, while if the aim was activation, perceived activation increased to greater extent; this increase was even more marked when participants had listened to music in order to achieve that aim. These findings could be interpreted in the way that a conscious choice of an aim plays an important role for the beneficial effects of music or combined musical- and language-based means. Although this is an interesting finding with potential implications for the clinical use of music and combined musical- and language-based means, our study was not designed to unravel the underlying mechanisms by which the choice of an aim impacts the effects on stress, relaxation and activation. Expectancy effects might play a role in this context. Furthermore, it is possible that people select different types of music to achieve different aims, e.g., slow music for relaxation and arousing music for activation. The same might apply to the choice of background music for language-based means. Furthermore, in our study, the choice of which music and combined musical and language-based means were offered to the participants depended on the choice of aim (if the aim was to relax, participants could choose from different relaxing pieces of music, exercises and texts; if they wanted to become activated, the proposed selection contained more energizing music and exercises). This means that we cannot clearly distinguish between the effects caused by music with different musical characteristics and such caused by participants’ intention to relax or to become activated. Future studies should take appropriate measures to shed light on the possible mechanisms underlying the effect of an aim of listening to musical means, language-based means, or combined means by obtaining data on musical characteristics (as we suggested in section “Stress Reducing Effects of Musical Compared to Language-Based or Combined Means**”**). This will be discussed in more depth in the following section.

### Strengths and Limitations

Since the app and the device used to measure ESR were originally designed and tested for commercial use, they had to be easy to handle and the burden for participants had to be kept low which resulted in certain limitations. We were able to measure electrodermal activity in an ambulatory setting and thus gained insight into sympathetic activity before and after listening to music, language-based, or combined means in an ambulatory setting. However, we only measured short periods of electrodermal activity before and after using the app. Future research should therefore employ an approach allowing measurements throughout listening to the means in order to gain knowledge about SNS activity during the process of listening. Furthermore, even though we could not take into account further means of stress reduction, such as yoga, exercise or reading a book, we still included a great variety of musical, language-based and combined stimuli yielding a higher generalizability than most laboratory studies. Moreover, there was no option to select an aim in mode 3 (language) because the tracks provided within this mode were too diverse to assign them to either the aim of relaxation or activation. Still, we were able to investigate effects of different musical and combined means as well as aims and, as an advancement with regard to previous studies, the interactions between means and aims. Another point of importance is that we were not able to control for the impact of musical characteristics which are known to determine the degree to that people perceive music as relaxing or arousing as well as stress-reducing, like tempo, rhythm and loudness. As noted in section “The Role of the Aim,” participants were provided with different musical pieces (which differed with regard to their characteristics) depending on their choice of aim, which means that we cannot clearly distinguish between the effects caused by music characteristics and such caused by participants’ intention to relax or to become activated. Moreover, comparability of the modes “music” and “music and language” is limited, as different genres of music (and thus music with different characteristics) were provided within these modes. Furthermore, background noise was not recorded but may have an effect on how well people can attend to and thus benefit from the means offered by the app. In the present study, however, it was not possible to obtain data on the abovementioned music characteristics or background noise. Follow-up studies should therefore consider using approaches that allow to track the music or language-based means allowing to derive their characteristics. Linnemann et al. (2018) for example achieved this by using the app “Simple Last.fm Scrobbler.” Future studies should also consider to control for background noise, which could be monitored by any application that is able to record sound, like iEar (Mehl, 2017).

Besides these limitations, the present study also includes several strengths: first, to the best of our knowledge, this is the first study to systematically compare the stress-reducing effects of musical and non-musical stimuli in an ambulatory setting. Second, we were able to conduct our investigation in the context of the participants’ work life. In our opinion, it is crucial to test whether means of stress reduction are actually effective in such settings, as work is a major source of stress and accordingly there is a greater need for stress relief during work hours. Third, in contrast to many other studies on stress-reducing effects of music, which comprised samples of mildly stressed university students, our sample consisted of highly stressed employees working in elevated positions. This made it possible to investigate whether previous findings on stress-reducing effects of musical and language-based means as well as the combination of both hold up under high-stress conditions. Still, as our sample mainly consists of highly-stressed individuals, generalizability of our findings is somewhat limited. Future studies could overcome this by including a group of highly stressed and one of moderately stressed individuals. Furthermore, it might be useful to not only compare different levels of stress experience but also to investigate whether an individual’s resiliency or susceptibility to stress impacts how well they can make use of the different means of stress reduction. Therefore, resiliency should be measured and controlled for. Despite this limitation, the setting, sample, and the aforementioned high variety of stimuli participants could choose from in our study, yield high ecological validity.

### Conclusion

Our findings suggest that in an ecologically valid setting listening to musical means, language-based means and a combination of both are equally effective in reducing stress and promoting relaxation. Music however, seems to be a more popular choice or more easily applicable in everyday life than the other means. Furthermore, music seems to be superior with regard to becoming energized, at least in comparison to combined musical and language-based means, making it a valuable strategy for recovering from stress and for replenishing one’s energy reserves. Moreover, our findings showed that the effect of listening to music or a combination of music and language-based means might depend on the aim one wants to achieve, even though further studies are needed to clarify whether this is indeed an effect of the intention to pursue a certain aim or an effect of specific musical characteristics. These findings suggest that music, language-based means of stress reduction, and combinations of both are valuable resources for promoting health. As not only musical but also language-based means of stress reduction and combinations of both are easily available *via* smartphone apps, they can be readily integrated into everyday life and work life. In particular, organizations and employees should be made aware of this cost-effective and easily applicable method of promoting health in the workplace. Furthermore, people should be encouraged to listen to music or to a combination of music and language-based means with the explicit aim of achieving relaxation in order to improve its stress-reducing and relaxing effects, and to consciously listen to music with the aim of activation in order to increase its energizing effect. In conclusion, it seems that apart from the means we use, the aim we want to achieve with it may be of importance. Future studies should apply online measurements of electrodermal activity or other ANS markers, in order to obtain more conclusive findings on differences in ANS activity between different means of stress reduction and different aims for using these. Additionally, markers of HPA axis activation should be used to assess physiological aspects of stress more broadly. Features of music such as tempo, rhythm, or loudness, as well as induced emotions should also be controlled for in future studies.

## Data Availability

The raw data supporting the conclusions of this manuscript will be made available by the authors, without undue reservation, to any qualified researcher.

## Author Contributions

AW-L, MK and UN designed the study. MK developed and performed the statistical analysis in conjunction with WS. MK wrote the first draft of the manuscript. All authors interpreted the data, reviewed and edited the manuscript, and approved the final version of the manuscript.

## Conflict of Interest Statement

The authors declare that the research was conducted in the absence of any commercial or financial relationships that could be construed as a potential conflict of interest. The reviewer CR declared a shared affiliation, though no other collaboration, with one of the authors, UN, to the handling Editor.
